# The Programme of the Saturday Fund

**Published:** 1891-06-27

**Authors:** 


### PASSING TOPICS.

### THE PROGRAMME OF THE SATURDAY FUND.

Mr. R. B. D. Acland is the Chairman of the Hospital Saturday Fund, and, as such, appears to have offered himself as a witness before the Lords' Committee, no doubt brimming over with good intentions and fully persuaded that he had something to say which was worth recording. True, we have Mr. Acland's own statement that he "does not profess to speak as an authority upon hospital management," and that he regards himself "as an apprentice on that at present," but as nothing is more commendable or uncommon than modesty, we will not be captious enough to inquire why, under such circumstances, he should come forward at all, nor will we deal too severely with the curious errors as to fact with which his evidence abounds.

We take it there are two classes of witnesses—those who go to give evidence because they must, and those who go because they choose—and no doubt, apprentice though he be, the Chairman of the Saturday Fund has no alternative but to say something. It is in his representative capacity that Mr. Acland acquires importance, and if his evidence possessed no other feature, it would be interesting as a manifesto of the aims and objects of the promoters of the Saturday Fund. We do not pretend that there is any startling novelty in the programme Mr. Acland spreads before us. We have known for a long time that the authorities of the Fund are bent upon exacting a *quid pro quo* in return for any assistance they render, and although we might have hoped at one time that worthier notions would ultimately prevail, we fear that the traditions of the Fund are too much interwoven with huckstering aims and pettifogging calculations to permit of free play to anything in the nature of just and generous sentiment. The dominant desire of the Hospital Saturday Fund is to drive a hard bargain with the hospitals, and to obtain favours wholly out of proportion to the scanty assistance it renders. Nay, more, it desires in return for its meagre contributions to pose as the censor of the institutions, to sap their independence and even to alter their character. The hospitals are no longer to be temples of benevolence; they are to be ministering servants of the working classes.

The evening attendances of the Physicians and Surgeons and the representation of the working class element upon the Boards of Hospitals are among the aims of this radical body, and, in short, the great hospitals of London are to be brought by the aid of the Saturday Fund pretty much into the position of medical clubs, with this difference, that the very utmost expectation of the authorities is to contribute 1s. in every 14s. of the expenditure, and apparently they believe the benevolent public will still go on supplying the remainder as if nothing had happened.

As to the improvement in management which the Saturday Fund apprentices expect to bring about, we have no faith in it, but were our belief in the efficacy of Saturday Fund control fifty times more robust than it is, we should still consider it pertinent to inquire what reason there is in the claim of working class representatives to meddle with the management of institutions whose work is virtually a free gift to their order?

The character and constitution of our hospitals may or may not call for the introduction of certain reforms. But we take exception to the preposterous self-sufficiency of the Saturday Fund authorities and we tell them frankly that the opinion of many hospital managers is that their help is not worth the price they are ambitious to obtain for it. Whenever the hospitals are compelled to sell their birthright, the Saturday Fund spokesmen may rest assured that better terms will be forthcoming than they are in a position to offer.

Agricola.

